# New Record of the *Hueberia* from the Posongchong Formation, Pragian Stage, Lower Devonian Series in the Bainiuchang Area, Southeastern Yunnan, China

**DOI:** 10.3390/life15111663

**Published:** 2025-10-24

**Authors:** Yukai Hu, Shitao Zhang, Liurunxuan Chen, Xianchao Chen, Shangyunzhi Xiao, Haonan Yin, Ruohan Zuo, Tao Wang, Xiaoqi Yang

**Affiliations:** 1 Faculty of Land and Resources Engineering, Kunming University of Science and Technology, Kunming 650093, China; digutisi@163.com (Y.H.); chenx1anchao@163.com (X.C.); 13550306625@163.com (S.X.); yinhn@live.com (H.Y.); zzzz_rh@163.com (R.Z.); w13934612450@163.com (T.W.); 2 Yunnan Institute of Geological Sciences, Kunming 650051, China; 3 Analytic & Testing Research Center of Yunnan, Kunming 650093, China; yxqgzx0917@163.com; 4 Research Center for Analysis and Measurement Kunming University of Science and Technology, Kunming 650093, China

**Keywords:** Early Devonian, Posongchong Formation, vascular plants, *Hueberia*, Taphonomy

## Abstract

The Posongchong Flora is an important window for understanding early vascular plant evolution. During a recent investigation of the Feigucun Section in the Bainiuchang area, Southeastern Yunnan, the author’s team discovered a large number of plant fossils from the Posongchong Formation of the Pragian Stage, Lower Devonian Series. Among the fossils collected in this investigation, there are six morphologically distinct drepanophycalean fossils, characterized by irregular dichotomous branching forming clustered stems, with the surface of the stems densely covered with spirally arranged falcate leaves. After comparing with coeval plant fossil records, it was found that the above characteristics are consistent with the generic characteristics of Hueberia fossils found in the Posongchong flora, indicating that these specimens should be assigned to the genus Hueberia. The team compared the newly discovered Hueberia fossils with the previously described *Hueberia zhichangensis*. The comparison revealed that the *Hueberia* specimens collected in the Bainiuchang area have thicker stems, longer intervals between successive branchings, and certain differences in leaf morphology from *Hueberia zhichangensis*. Therefore, the team considers that there are significant differences between it and *Hueberia zhichangensis* in morphology, geographical distribution, and other aspects, and there is sufficient reason to erect a new species, *Hueberia bainiuchangensis* sp. nov. The discovery of this species enriches our understanding of the species diversity of the genus *Hueberia*, expands the geographical distribution range of the Posongchong Flora, further corroborates that the Southeastern Yunnan region had extremely high biodiversity during the Pragian Age of the Early Devonian, and provides new clues for the study of Early Devonian plant evolution.

## 1. Introduction

As one of the most important primary producers, terrestrial plants are a key component of the Earth system and the foundation of terrestrial ecosystems [1]. Terrestrial plants originated in the Ordovician and formed the earliest forests by the Devonian [2]. The origin and radiation of terrestrial plants represent a major event in Earth’s life history, profoundly transforming the Earth’s surface system and serving as an important milestone in the evolution of Earth’s habitability [3].

The Early Devonian was a critical period in the evolution of terrestrial ecosystems, during which low-growing early vascular plants underwent their first major radiation, with rapidly increased diversity [4]. This not only completely reshaped terrestrial landscape patterns but also laid the ecological foundation for the subsequent animal colonization of land. During this period, Southeastern Yunnan was located on the southwestern margin of the Yangtze Plate and contained abundant and diverse fern fossils. Among the relevant floras discovered in Southeastern Yunnan, the most typical is the Posongchong Flora, which is regarded as a window for research on evolution and diversity of early land vascular plants [5]. It is dated to the Late Pragian of the Early Devonian [6,7,8,9,10], with a typical section located in Zhichang Village, Wenshan City, Yunnan Province, China [11].

Among them, the *Hueberia* was first discovered in this plant fossil assemblage, and prior to this study, only one species—*Hueberia zhichangensis*—had been identified [12,13]. Our team recently conducted an investigation of the Posongchong Formation section west of Feigu Village in the Bainiuchang area of Southeastern Yunnan. This investigation revealed that the section preserves abundant and diverse plant fossils, which are relatively well-preserved, mostly as carbonaceous films or impressions. Among the fossils collected in this survey, one morphologically distinctive type is characterized by clustered stems formed by irregular dichotomous branching, with the stem surface densely covered by spirally arranged falcate leaves. These features conform to the generic characteristics of *Hueberia* [12], indicating that these specimens should be assigned to the *Hueberia*. Compared with *Hueberia zhichangensis*, they have thicker stems, longer intervals between successive branches, and certain differences in leaf morphology, etc. Therefore, this is erected as a new species of *Hueberia*—*Hueberia bainiuchangensis* sp. nov.

## 2. Materials and Methods

**Figure 1:**
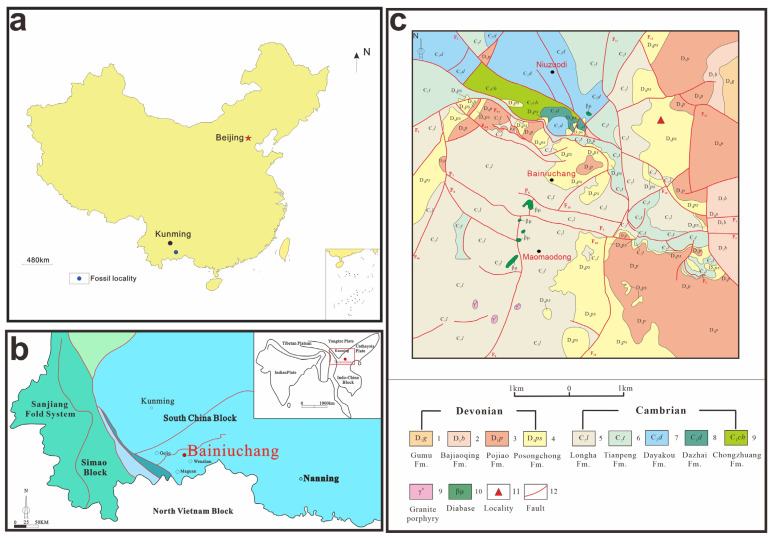
(**a**) Fossil locality; (**b**) tectonic position of Bainiuchang; (**c**) geological map of the study area.

**Figure 2:**
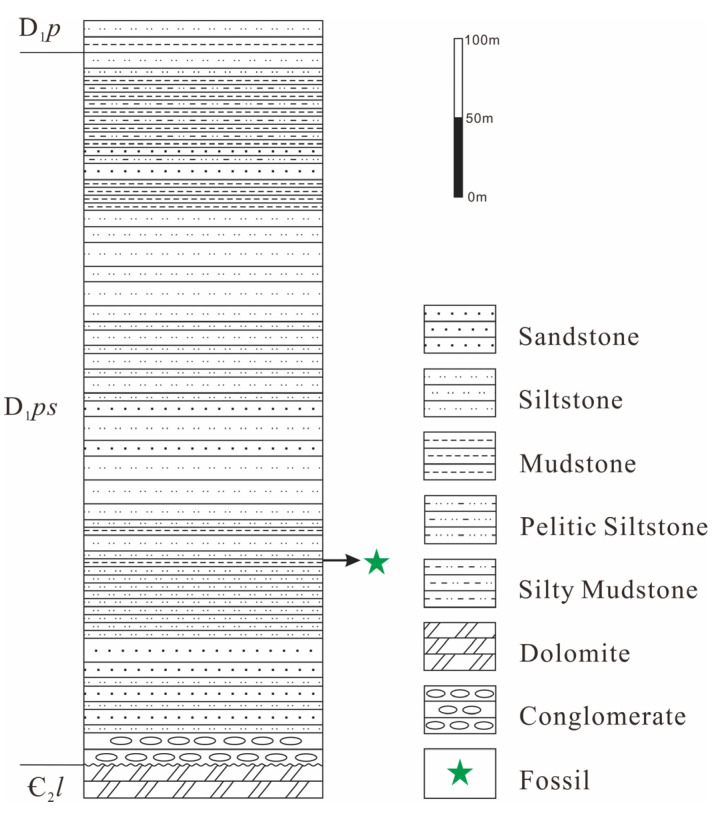
Lithology column chart of strata.

The materials in this study were collected from the Posongchong Formation section located 1.2 km west of Feigu Village, Wenshan Prefecture, Yunnan Province (Figure 1). This section is in angular unconformity with the underlying Middle Cambrian Longha Formation limestone and dolomite, and the overlying strata are the Lower Devonian Pojiao Formation mudstone and siltstone. The section is approximately 446 m thick, with specimens collected at 129 m thickness. The lithology column chart of the strata is presented in Figure 2. The lithology is gray and yellowish-gray medium-thin bedded siltstone and mudstone. The collection coordinates are 103°47′39.9″ E, 23°29′39.3″ N.

A total of 6 new specimens of the *Hueberia* were collected from the Feigu Section, numbered KUSTPSCHS401 to KUSTPSCHS406. All specimens are housed in the Geological Museum of Kunming University of Science and Technology. The newly collected *Hueberia* specimens are mainly preserved as impression fossils, with some specimens showing carbon film preservation characteristics, and the preserved parts of the fossils are predominantly stems.

The collected specimens were photographed using a digital camera and numbered for archiving. To further observe the structure of the fossil specimens, the author team conducted microscopic observation and photography of the specimens using a LEICA S6D stereomicroscope at the Faculty of Land and Resources Engineering, Kunming University of Science and Technology. Additionally, specimen KUSTPSCHS403, which is well-preserved with size and flatness meeting the requirements for SEM analysis, was selected for analysis. Uncoated samples were analyzed using a Hitachi SU3900 Scanning Electron Microscope (SEM) (Hitachi, Tokyo, Japan) and an OXFORD ULTIM MAX 40 Energy Dispersive Spectrometer (EDS) (Oxford Instruments, Abingdon, United Kingdom) at the Analysis and Testing Research Center of Kunming University of Science and Technology; the SEM employs an acceleration voltage of 5.00 kV, the EDSmapping employs an acceleration voltage of 15.00 kV. Furthermore, whole-rock major, trace, and rare earth element (REE) analyses were performed on the host rocks of the fossil specimens. These analyses were conducted by China Metallurgical and Geological Bureau Shandong Bureau Group Testing Co., Ltd. (Jinan, China). The major element analysis was carried out using an XR-PFX-04U X-ray Fluorescence (XRF) spectrometer (Thermo Fisher Scientific, Waltham, MA, USA) (serial number YQ273). A mixture of lithium tetraborate and lithium metaborate was used as a flux, with ammonium nitrate as the oxidizing agent and a small amount of ammonium bromide as the mold release agent. The mass ratio of test sample to reference sample was 1:10. Samples were melted at 1150 °C in a muffle furnace to form glass specimens, which were then analyzed using an XRF spectrometer. Trace element and rare earth element analyses were performed using an Agilent 8900 triple quadrupole Inductively Coupled Plasma Mass Spectrometer (ICP-MS) (Agilent Technologies, Santa Clara, CA, USA) (serial number YQ265), in accordance with the standard GB/T 14506.28-2010 [14]. Dissolved in a sealed digestion vessel with hydrofluoric acid and nitric acid, the samples underwent hydrogen fluoride evaporation via an electric heating plate. Subsequently, they were dissolved in aqua regia under sealed conditions. After dilution, trace elements and rare earth elements were determined using the external standard method with ICP-MS.

## 3. Description and Comparison

### 3.1. Description

Among the 6 collected fossil specimens, KUSTPSCHS401 to KUSTPSCHS404 are well-preserved, preserving relatively long stem portions with visible distinct structures on the surface, while the other two are poorly preserved, only stem fragments less than 10 mm in size. The four well-preserved specimens are described in detail as follows:

#### 3.1.1. KUSTPSCHS401

**Figure 3:**
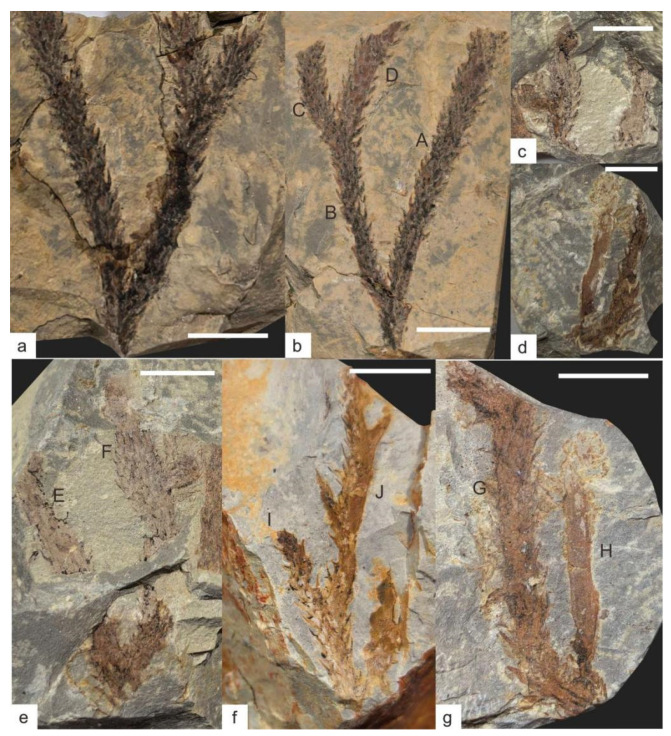
*Hueberia bainiuchangensis* from the Lower Devonian Posongchong Formation of Yunnan, China: (**a**,**b**) KUSTPSCHS401; (**c**,**e**) KUSTPSCHS402; (**d**,**g**) KUSTPSCHS403; (**f**) KUSTPSCHS404. Scale bar = 10 mm. The letters (A–J) in the Figure 3 represent branch codes.

**Figure 4:**
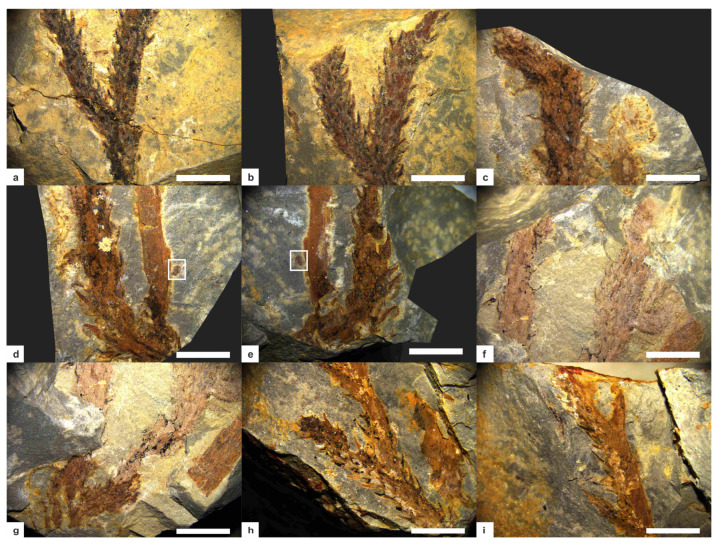
*Hueberia bainiuchangensis* from the Lower Devonian Posongchong Formation of Yunnan, China. Enlargement of (Figure 3): (**a**,**b**) two branches of KUSTPSCHS401, scale bar = 5 mm; (**c**–**e**) KUSTPSCHS403, (**c**) is the bend of the suspected branch, (**d**,**e**) are the positions of the branches, and the white frames are the sporangium, scale bar = 5 mm; (**f**,**g**) KUSTPSCHS402, (**f**) is the position of stem enlargement, and (**g**) is the position of branching, scale bar = 5 mm.; (**h**,**i**) KUSTPSCHS404, (**h**) is the first branch and (**i**) is two small branches with opposite directions, scale bar = 5 mm.

This specimen mainly preserves the stem, Figure 3a,b, Figure 4a,b, with two dichotomous branches; the apex and base are not preserved. The total length of the specimen is 47 mm. The first branch occurs 9 mm above the base; it is divided into A and B branches, with a branching angle of approximately 35°. The stem width at the branching point is 3.9 mm, tapering downward, with the narrowest stem width of 2.8 mm at the bottom; this part of the stem has falcate leaves approximately 2 mm long.

Branch A measures 38 mm in length, gradually thickening from base to tip with a minimum width of 2.5 mm and maximum width of 3.7 mm. Except for the 0–6 mm section on one side where no falcate leaves are observed, the remaining parts are covered with falcate leaves. The leaves are falcate, tapering from the base to the apex, slightly curved inward, forming an angle of approximately 30–60° with the stem. They are mostly about 2 mm in length (maximum up to 2.7 mm) and 0.1–0.7 mm in width. Based on surface protrusions and depressions, the falcate leaves are spirally arranged, with approximately 6–8 leaves per whorl and a whorl spacing of about 1 mm.

Branch B gradually thickens from its base, with a minimum width of 2.8 mm and a maximum width of 4.2 mm. It dichotomously branches again at 23.5 mm along this branch; it is divided into C and D branches, with a branching angle of approximately 30°. Branch C is 9.6 mm long and Branch D is 12.5 mm long; both branches have relatively uniform widths—C width about 2.5 mm and D width about 2.8 mm. Branches B, C, and D are all covered with falcate leaves, with leaf morphology and distribution similar to those of Branch A: angles of 30–60° with the stem, lengths of about 2 mm (maximum up to 2.5 mm), and widths of 0.1–0.7 mm.

#### 3.1.2. KUSTPSCHS402

This specimen mainly preserves the stem, Figure 3c,e, Figure 4f,g, with two dichotomous branches; the apex and base are not preserved. The total length of the specimen is 34.5 mm. Dichotomous branching occurs 4.5 mm from the base upwards; it is divided into E and F branches, with a branching angle of approximately 30°. The stem width at the branching point is 5.8 mm, and this part of the stem has falcate leaves approximately 2 mm long.

Branch E has a stem length of 22 mm, with a minimum width of 2.9 mm and a maximum width of 3.5 mm. It is poorly preserved, with no distinct structures visible on the surface, except for one falcate leaf 2 mm long, 0.1–0.5 mm wide.

Branch F has a stem length of 30 mm, with a minimum width of 2.9 mm and a maximum width of 4.2 mm. It is entirely covered with falcate leaves, with leaf shape and distribution similar to other specimens: lengths of about 2 mm (maximum up to 3.2 mm), whorl spacing of about 1 mm, and angles of 30–40° with the stem. Dichotomous branching occurs at 18 mm along this branch, with a branching angle of approximately 90°, and stem enlargement is visible at the branching point. The resulting branch is poorly preserved, 6.1 mm long, 2.0–3.2 mm wide, and not covered with falcate leaves.

#### 3.1.3. KUSTPSCHS403

This specimen mainly preserves the stem, Figure 3d,g, Figure 4c–e, with one dichotomous branch; the apex and base are not preserved. The specimen is 39 mm long in total, and a bifurcation occurs at about 3 mm from the bottom upward into two branches, G and H, with a branching angle of approximately 30°. The stem width at the branching point is 4.9 mm, and this part of the stem has no falcate leaves.

Branch G has a stem length of 36 mm, with a minimum width of 2.4 mm and a maximum width of 4.2 mm at 5.8 mm along the branch, where the stem exhibits a distinct curvature. The stem curves again at 30 mm along this branch; based on preservation, this is a branching point, but the other branch is not preserved. It is entirely covered with falcate leaves, with leaf shape and distribution similar to previous ones: angles of 30–50° with the stem, lengths relatively longer (mostly over 2 mm, maximum up to 3.2 mm), widths of 0.1–1 mm, and leaves more sparsely distributed with a whorl spacing of approximately 1.5 mm.

Branch H has a stem portion length of 19 mm, with a minimum width of 2.3 mm and a maximum width of 3.2 mm. Its surface is relatively clean and not covered with falcate leaves. A sporangium is visible 6.2 mm from the bottom upwards along this branch, with a vertical length of 1.1 mm and a horizontal width of 0.85 mm.

#### 3.1.4. KUSTPSCHS404

This specimen mainly preserves the stem, Figure 3f, Figure 4h,i, with three dichotomous branches; the apex and base are not preserved. The total length of the specimen is 39.5 mm. Dichotomous branching occurs 12.7 mm from the base upwards, it is divided into I and J branches, with a branching angle of 35°. The stem width of this part is 2.8 mm, and 4 mm at the branching point, with falcate leaves 2–2.8 mm long.

Branch I is 9.7 mm long, with a relatively uniform width of approximately 2.2 mm. Its surface is covered with falcate leaves about 2 mm long, with shape and distribution similar to other specimens.

Branch J is 26.8 mm long and 2.2 mm wide. Falcate leaves are visible on the left side, 2–3 mm long, with a shape similar to other specimens; no falcate leaves are preserved on the right side and surface. Dichotomous branching occurs at 10.5 mm and 21 mm along this branch, with lengths of 6.8 mm and 5.6 mm, respectively, branching in opposite directions, poorly preserved, with no distinct falcate leaves.

### 3.2. Comparison

**Figure 5:**
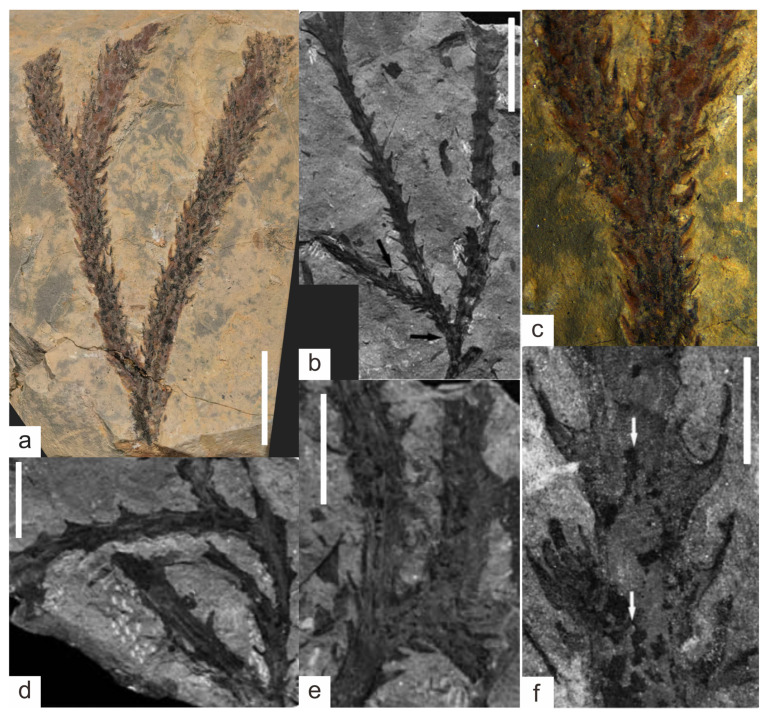
Comparison of *Hueberia zhichangensis* and *Hueberia bainiuchangensis* (Figure 5b,d–f of *Hueberia zhichangensis* are from Xue, 2013 [13]). (**a**,**b**) show the differences in stem thickness between the two, scale bar = 10 mm. (**c**,**f**) show the differences in leaves between the two. Most of the leaf morphology of the specimens in this paper is as shown (**c**), whereas most of the leaf morphology of *Hueberia zhichangensis* is as shown (**f**), (**c**) scale bar = 5 mm. (**f**) Scale bar = 2 mm, (**d**,**e**) show the dense branching (**d**) and K-type branching (**e**), which was found in the specimens of this paper, Scale bar = 10 mm.

We conducted a detailed comparison between the plant fossil specimens collected in this study and the previously discovered plant fossils from the Posongchong Formation [15], and found that they are most similar to the *Hueberia*. The specimens in this study are characterized by clustered stems formed by irregular dichotomous branching, with the stem surface densely covered by spirally arranged falcate leaves. This feature conforms to the generic diagnosis of *Hueberia*; therefore, these specimens should be assigned to the genus *Hueberia*. Among the currently reported plant fossils, only *Hueberia zhichangensis*, discovered in the Zhichang section of Wenshan City, has been assigned to *Hueberia*. We compared the *Hueberia* fossils discovered in this study with the previously described *Hueberia zhichangensis* and found obvious differences between them (Table 1): first, in terms of stem thickness (Figure 5a,b), the specimens in this study have significantly thicker stems, with a stem width generally greater than 2 mm, typically around 3 mm, whereas the stem width of *H. zhichangensis* is generally less than 2 mm, with a maximum width of only 2.8 mm. Second, in terms of leaf morphology (Figure 5c,f), the present specimens exhibit certain similarities to it; however, the base is rarely observable in most leaves of the present specimens, whereas the leaves of *H. zhichangensis* typically exhibit a distinct base. Furthermore, the leaves of the present specimens mostly show uniform contraction from the base upwards, whereas the leaves of *H. zhichangensis* mostly exhibit rapid contraction from the base upwards. Third, in terms of branching type, the intervals between successive branches in the specimens of this study are generally longer, ranging from 10 mm to a maximum of 30 mm, showing relatively simple dichotomous branching (Figure 5a), whereas H. zhichangensis frequently exhibits more densely spaced successive branches (Figure 5d), forming K-type and H-type branching (Figure 5e), and no lateral buds were found in the specimens of this study. In addition, the length (1.7–3.2 mm), width (0.1–1 mm), and whorl spacing (1–1.5 mm) of the falcate leaves in the specimens of this study are also greater than those of H. zhichangensis (0.7–2.3 mm, 0.1–0.5 mm, and 1–1.2 mm, respectively). In summary, our team believes that there are significant differences between this taxon and *Hueberia zhichangensis* in terms of morphology and geographical distribution, and there is sufficient reason to establish it as a new species. Based on the discovery locality of this species in the Bainiuchang area of southeastern Yunnan, it is named *Hueberia bainiuchangensis*.

**Table 1:** Comparison of *Hueberia zhichangensis* and *Hueberia bainiuchangensis* (modified from Xue, 2013 [13]).

	*Hueberia* *zhichangensis*	*Hueberia* *zhichangensis*	*Hueberia* *bainiuchangensis*
STEMS			
Branching pattern	Frequently isotomous	Frequently isoanisotomous	Dichotomus
Rhizome width (mm)	1.3–1.8	1.1–3.4	-
Aerial stem width (mm)	1.3–1.8	0.9–2.8	2.0–4.9
BUDS			
Distribution	-	Irregular	-
Width (mm)	-	0.7–1.2	-
Length (mm)	-	0.7–2.6	-
ENATIONS or MICROPHYLLS			
Arrangement	Helical; 6–8 per gyre	Helical; 6–8 per gyre	Helical; 6–8 per gyre
Morphology	Falcate enations	Falcate microphylls	Falcate microphylls
Width (mm)	0.6–1.0	0.1–0.5	0.1–1
Length (mm)	0.9–1.6	0.7–2.3	1.7–3.2
Height of bases (mm)	-	0.6–1.6	-
SPORANGIA	-		
Outline in face view	-	Rounded	Rounded
Size (mm)	-	0.6–0.9	0.85–1.1
REFERENCES	Yang et al., 2009 [12]	Xue, 2013 [13]	This study

**Figure 6:**
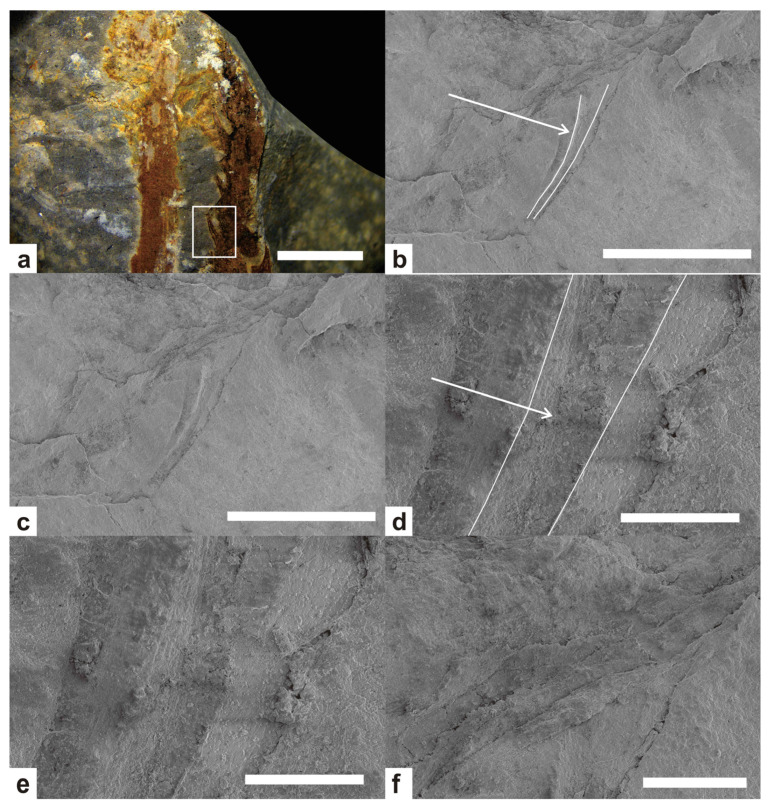
SEM photographs of *Hueberia bainiuchangensis*: (**a**) scanning position of *Hueberia bainiuchangensis*, the white frame is scanning position, scale bar = 5 mm. (**b**) SEM image of *Hueberia bainiuchangensis*, the arrow points to the vascular structure, scale bar = 2 mm. (**c**) (Figure 4b) unannotated version, scale bar = 2 mm. (**d**) Enlargement of the leaf position in (Figure 4b), the arrow points to the vascular structure, scale bar = 200 μm. (**e**) (Figure 4d) unannotated version, scale bar = 200 μm. (**f**) Enlargement of the stem position in Figure 4b, scale bar = 500 μm.

The falcate leaves of Hueberia were initially described as thalli in the original study [12]. In subsequent studies [13], vein structures were found through coalified residues in the leaves, and combined with other features, they were recognized as leaves. In this study, we photographed the falcate leaves using a scanning electron microscope and found obvious vascular structures in the leaves (Figure 6b,d), which provide strong evidence that the falcate protrusions are leaves, confirming that they are indeed leaves. The central vascular bundle of the falcate leaves in Hueberia is distributed in the middle of the leaf, slightly biased towards the back of the leaf, with a width slightly greater than that of both sides. It curves inward similarly to the leaf and tapers as the leaf tapers.

### 3.3. Systematic Paleontology

*Hueberia* Yang et al., 2009, [12]

*Hueberia bainiuchangensis* sp. nov.

Etymology: Referring to the Bainiuchang area, Yunnan Province.

Diagnosis: Stem 2.0–4.9 mm wide, with dichotomous branching; branching angle 30–90°. Stem mostly covered with falcate leaves. Leaf outline falcate, base indistinct, leaves uniformly tapering from base to apex. Length 1.7–3.2 mm, width 0.1–1 mm; angle with stem 30–60°, mostly less than 45°, spirally arranged, 6–8 per whorl; whorl interval approximately 1.0–1.5 mm. Sporangia lateral, rounded in shape, 0.85–1.1 mm in diameter.

Holotype: KUSTPSCHS401

Paratypes: KUSTPSCHS402, KUSTPSCHS403, KUSTPSCHS404

Type locality: Bainiuchang, Yunnan Province, China

## 4. Discussion

The Posongchong Flora, as one of the most representative fossil repositories of Early Devonian terrestrial vascular plants, has attracted significant attention from the academic community and extensive research since the 20th century [16]. To date, at least 29 genera and 38 species of fern fossils have been discovered in the Posongchong Formation, with at least 18 genera first found in this stratigraphic unit [17]. Among these numerous genera and species, it is evident that a rich and diverse assemblage of terrestrial vascular plants inhabited the southeastern Yunnan region during the Early Devonian. Notably, among the currently known taxa, only four genera—*Baragwanathia*, *Drepanophycus*, *Halleophyton*, and *Hueberia*—are assigned to the Drepanophycales [18,19], indicating that Drepanophycales plants are a relatively rare component of the Posongchong Flora. Furthermore, the genera *Halleophyton* and *Hueberia* have thus far only been discovered in the Posongchong Formation of southeastern Yunnan [18], suggesting that these two genera may represent an endemic taxon of the southeastern Yunnan region. The fossil specimen discovered in this study is identified as a new species of *Hueberia*. The discovery of this species not only further increases the biodiversity of Drepanophycales and its proportion in the Posongchong Formation but also expands the geographical distribution range of the Posongchong flora. It further supports the conclusion that southeastern Yunnan exhibited extremely high biodiversity during the Pragian Stage of the Early Devonian and provides new insights into the study of Early Devonian plant evolution. This indicates that the Posongchong flora still holds research potential after years of study, with possible undiscovered plant taxa warranting further investigation.

The fossil specimens were collected from siltstones and mudstones of the Posongchong Formation, which are extremely fine-grained sediments, indicating that the specimens were deposited in a low-energy sedimentary environment. The preservation state is generally good: most stems and falcate leaves are clearly visible, with only a few specimens showing slight damage, a feature suggesting that the fossils did not undergo long-distance transportation. Yang (2009) [12], when discussing the burial environment of *Hueberia zhichangensis*, noted that the well-preserved slender branches and extremely small phyllids, the vertical posture of branches, undisturbed branching angles, and fine-grained matrix indicate autochthonous burial or short-distance transportation. The specimens in this study exhibit similar preservation characteristics, suggesting they were also autochthonously buried or transported over short distances.

**Figure 7:**
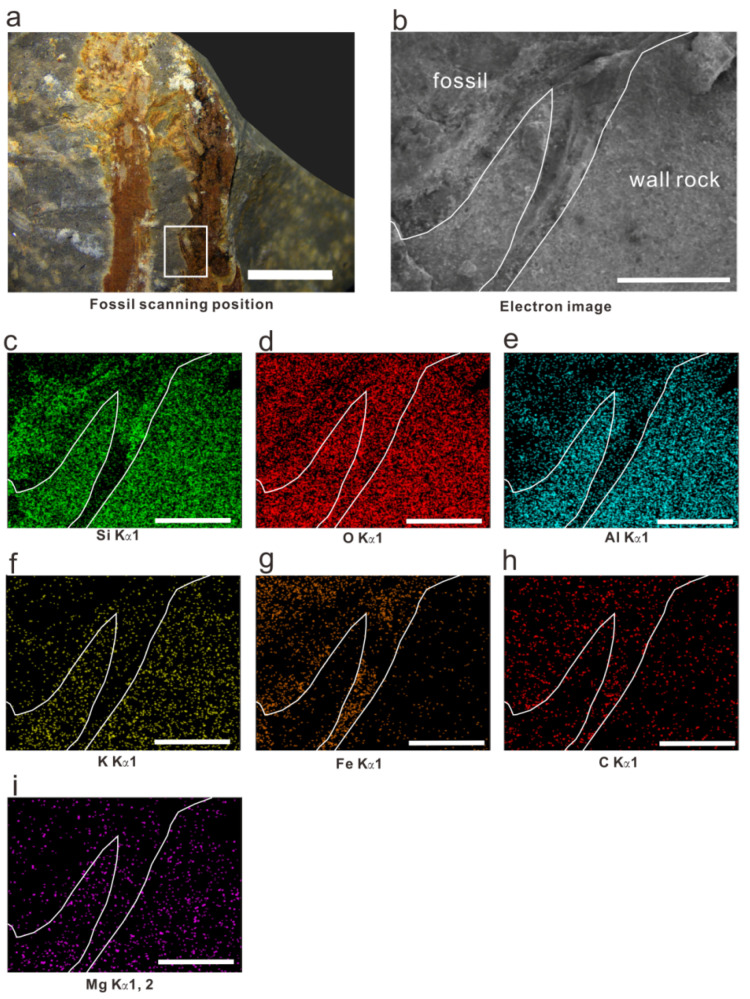
SEM-EDS mapping results of *Hueberia bainiuchangensis*: (**a**) scanning position of the fossil, the white frame is scanning position; scale bar = 5 mm; (**b**) electron image of the fossil; scale bar = 1 mm; (**c**–**i**) distribution results of Si, O, Al, K, Fe, C, Mg elements; scale bar = 1 mm.

We conducted SEM-EDS mapping on the falcate leaves of the new *Hueberia* specimens; the distribution patterns of Si, O, Al, K, Fe, C, and Mg are shown in Figure 7. Si is less distributed in the fossil part than in the host rock but is somewhat enriched at the edges of stems and the bases of leaves. O is generally uniformly and densely distributed, with slightly lower concentrations at the edges of stems. Al shows significant distribution differences, being obviously enriched in the host rock and significantly more abundant there than in the fossil part. K is slightly enriched in the host rock and rarely distributed in the fossil part. Fe is rarely distributed in the host rock but somewhat enriched in the fossil part, with slightly lower concentrations at the edges of stems and the bases of leaves. C is more enriched in the fossil part, though the difference between the fossil and host rock is smaller than that of other elements. The distribution characteristics of Mg are the opposite of those of C. The uniform and dense distribution of O indicates that Si and metal elements mostly exist in the form of oxides in both the host rock and the fossil. X-rays can penetrate thin samples, and since the samples in this study are mainly preserved as impressions, the contents of Si, Al, K, and Mg in the fossil layer are lower than those in the rock matrix. This may be because they appear on the map due to X-ray penetration and are merely components of aluminosilicate sediments. The enrichment of C in the fossil part may be evidence of the preservation of residual organic matter, and the enrichment of Fe indicates that the sample underwent a certain degree of mineralization during the fossilization process.

To further reconstruct the paleoenvironment, we also conducted whole-rock major, trace, and rare earth element (REE) analyses on the host rocks of the fossils and used the analytical results to analyze redox conditions and Paleoclimate. The test results are shown in Table 2.

**Table 2:** Major, trace, and rare earth element test results.

Test Item	Unit	Test Result	Test Item	Unit	Test Result	Test Item	Unit	Test Result	Test Item	Unit	Test Result
K_2_O	%	4.52	V	μg/g	100	Cs	μg/g	28.8	Tm	μg/g	0.655
Al_2_O_3_	%	20.81	Mn	μg/g	64.2	Ba	μg/g	452	Yb	μg/g	4.26
CaO	%	0.07	Co	μg/g	4.43	B	μg/g	93.7	Lu	μg/g	0.638
TFe_2_O_3_	%	2.41	Ni	μg/g	19.1	La	μg/g	54.1	Hf	μg/g	5.72
MgO	%	0.904	Cu	μg/g	11.9	Ce	μg/g	110	Ta	μg/g	1.4
Na_2_O	%	0.233	Zn	μg/g	35.8	Pr	μg/g	12.6	W	μg/g	2.36
SiO_2_	%	64.41	Ga	μg/g	24.7	Nd	μg/g	50.7	Ti	μg/g	0.987
MnO	%	0.007	Rb	μg/g	193	Sm	μg/g	9.59	Pb	μg/g	31.4
P_2_O_5_	%	0.057	Sr	μg/g	68.9	Eu	μg/g	1.67	Bi	μg/g	0.511
TiO_2_	%	0.819	Y	μg/g	37.9	Gd	μg/g	8.6	Th	μg/g	24.3
Li	μg/g	78.5	Zr	μg/g	262	Tb	μg/g	1.32	U	μg/g	3.57
Be	μg/g	2.37	Nb	μg/g	19.2	Dy	μg/g	7.53	In	μg/g	0.099
Sc	μg/g	12.9	Mo	μg/g	0.412	Ho	μg/g	1.45			
Ti	μg/g	4842	Cd	μg/g	0.038	Er	μg/g	4.32			

The analysis of redox conditions using geochemical data primarily relies on the fact that the solubility of redox-sensitive elements in water is significantly controlled by the oxidation state of the water column; during changes in redox conditions, the occurrence state of redox-sensitive elements alters [20]. V system is a widely used method for assessing redox conditions [21,22]; in addition, REEs are also frequently used to reflect the redox conditions of paleowater columns [23,24].

For V system proxies, we calculated the values of Ni/Co, V/(V + Ni), and U/Th [25,26] for the samples. For REEs, we calculated the Ce_anom_ values [27,28] of the samples. The calculation formula [29,30] for Ce_anom_ is

(1) Ce_anom_ = log [3Ce_N_/(2La_N_ + Pr_N_)]

The samples were normalized using North American shale standard values [31]. The calculation results of V system proxies (Table 3) indicate that the samples were deposited in an oxidizing environment, while the Ceanom calculation results (Table 3) show that the samples have a slight negative Ce anomaly, indicating a weakly oxidizing environment [30]. Combined with other data, it is more likely that the deposition occurred in an oxidizing to weakly oxidizing environment at that time.

In the paleoclimate, we selected Sr/Cu as the discriminant index. Sr and Cu are important indicators of climate environment. Generally, the Sr content is often higher in an arid climate environment, while it is relatively lower in a humid climate environment [32]. The results (Table 3) show that it was a semi-arid climate environment at that time.

**Table 3:** Redox indicator and paleosalinity indicator.

Redox Indicator	RRs [30]	Result	Paleoclimate	RRs [33]	Result
Ni/Co	Oxidizing environment < 7 Anoxic environment > 7	4.31	Sr/Cu	Arid climate > 10 Semi-arid climate 5–10 Humid climate < 5	5.79
V/(V+Ni)	Oxidizing environment < 1.5 Anoxic environment > 1.5	0.84			
U/Th	Oxidizing environment < 1.25 Anoxic environment > 1.25	0.15			
Ce_anom_	Oxidizing environment < 0 Anoxic environment > 0	−0.03			

## 5. Conclusions

In this study, new plant fossil materials of the order Drepanophycales were discovered in the Bainiuchang area of the Posongchong Formation (Early Devonian) in Yunnan. Through detailed morphological comparison and systematic paleobotanical analysis, a new species of the *Hueberia, Hueberia bainiuchangensis*, is erected. SEM observation revealed that the falcate leaves possess obvious vascular structures, providing key evidence that the falcate projections of the genus *Hueberia* are leaves.

The discovery of the new species further enriched the species diversity of the Posongchong Flora and increased the proportion of Drepanophycales in this flora. Combined with the current records that the *Hueberia* has only been found in the Posongchong Formation of southeastern Yunnan, this supports the speculation that the genus may be an endemic taxon of the Early Devonian in southeastern Yunnan, providing new evidence for the extremely high plant diversity in southeastern Yunnan during the Pragian Stage of the Early Devonian.

Results of taphonomic analysis indicate that the fossil specimens underwent a certain degree of mineralization during the fossilization process, retained a certain amount of organic matter, not subjected to long-distance transport, and are in an autochthonous or parautochthonous (short-distance transport) state, deposited under conditions of semi-arid climate, low hydrodynamic energy, and oxidizing to weakly oxidizing environments.

In conclusion, the discovery of *Hueberia bainiuchangensis* not only provides new data for the evolution and taxonomy of the genus *Hueberia*, but also adds important clues to the studies of paleophytogeography, paleoenvironmental reconstruction, and the evolution of terrestrial vascular plants in southeastern Yunnan during the Early Devonian.

## Data Availability

The datasets presented in this article are not readily available because the data are part of an ongoing study or due to technical time limitations. Requests to access the datasets should be directed to [Y.K.H.].
